# Malaria chemoprevention and drug resistance: a review of the literature and policy implications

**DOI:** 10.1186/s12936-022-04115-8

**Published:** 2022-03-24

**Authors:** Christopher V. Plowe

**Affiliations:** University of Maryland School of Medicine, Baltimore, MD USA

## Abstract

Chemoprevention strategies reduce malaria disease and death, but the efficacy of anti-malarial drugs used for chemoprevention is perennially threatened by drug resistance. This review examines the current impact of chemoprevention on the emergence and spread of drug resistant malaria, and the impact of drug resistance on the efficacy of each of the chemoprevention strategies currently recommended by the World Health Organization, namely, intermittent preventive treatment in pregnancy (IPTp); intermittent preventive treatment in infants (IPTi); seasonal malaria chemoprevention (SMC); and mass drug administration (MDA) for the reduction of disease burden in emergency situations. While the use of drugs to prevent malaria often results in increased prevalence of genetic mutations associated with resistance, malaria chemoprevention interventions do not inevitably lead to meaningful increases in resistance, and even high rates of resistance do not necessarily impair chemoprevention efficacy. At the same time, it can reasonably be anticipated that, over time, as drugs are widely used, resistance will generally increase and efficacy will eventually be lost. Decisions about whether, where and when chemoprevention strategies should be deployed or changed will continue to need to be made on the basis of imperfect evidence, but practical considerations such as prevalence patterns of resistance markers can help guide policy recommendations.

## Background

After decades of dramatic reductions in malaria cases and deaths worldwide, progress toward malaria control and elimination had plateaued before the COVID-19 pandemic [1], and malaria cases and deaths rose in 2020 [2]. Further erosion of the recent gains in malaria control will lead to resurgences, at great cost to the health, lives, and economies of the world’s poorest countries [3]. Chemoprevention strategies, i.e., the use of anti-malarial medicines for prophylaxis and for preventive treatment, can be effective tools for malaria control and elimination, but the risks of resistance to anti-malarial drugs used for prevention and treatment must be mitigated and managed for momentum to be regained and sustained.

The chemoprevention strategies currently recommended by the World Health Organization (WHO) include intermittent preventive treatment in pregnancy (IPTp), intermittent preventive treatment in infants (IPTi), seasonal malaria chemoprevention (SMC), and mass drug administration (MDA) for the reduction of disease burden in emergency situations (Table 1). From the time that these chemoprevention strategies were first conceived, concerns have been raised both about their potential impact on the development and spread of drug resistance that might compromise the treatment efficacy of the drug classes used, and about the impact of drug resistance on the efficacy of different chemoprevention strategies.

**Table 1:** Definitions of Malaria Chemoprevention Strategies*

Intermittent preventive treatment in pregnancy (IPTp)	A full therapeutic course of anti-malarial medicine given to pregnant women at routine prenatal visits, regardless of whether the woman is infected with malaria
Intermittent preventive treatment in infants (IPTi)	A full therapeutic course of sulfadoxine-pyrimethamine delivered to infants in co-administration with DTP2/Penta2, DTP3/Penta3 and measles immunization, regardless of whether the infant is infected with malaria
Seasonal malaria chemoprevention (SMC)	Intermittent administration of full treatment courses of an anti-malarial medicine during the malaria season to prevent malarial illness. The objective is to maintain therapeutic concentrations of an anti-malarial drug in the blood throughout the period of greatest risk for malaria
Note: This intervention is recommended only for areas with highly seasonal malaria, where transmission occurs during a few months of the year	
Mass drug administration (MDA)	Administration of anti-malarial treatment to all age groups of a defined population or every person living in a defined geographical area (except those for whom the medicine is contraindicated) at approximately the same time and often at repeated intervals

^*^Definitions from WHO Malaria Terminology, last updated 2019[4]

### Measuring and monitoring resistance and efficacy

#### Clinical trials remain the gold standard for measuring and monitoring efficacy

As they have been developed and implemented, each chemoprevention strategy has been evaluated in complex prospective, controlled trials that typically randomly assign either individuals or clusters (e.g., villages or districts) to receive either the drug prevention regimen being tested, or an alternative regimen, or no preventive regimen. Because their primary outcome measures are affected by a number of host and parasite factors in addition to parasite resistance, efficacy trials do not directly measure drug resistance per se, but these prospective studies remain the gold standard for measuring the chemoprevention efficacy.

The efficacy of drugs used to treat malaria is monitored worldwide in single-arm clinical trials known as WHO Therapeutic Efficacy Studies (TES), which follow standardized protocols to provide direct evidence of drug efficacy to guide policy decisions [5]. Unlike these TES for routine monitoring of treatment efficacy, simplified protocols for routine monitoring of chemoprevention efficacy are not yet in use, although the WHO is presently developing such streamlined protocols.

Because anti-malarial drug resistance is considered paramount among the many host, parasite, pharmacological and other factors that affect the efficacy of anti-malarial drugs, methods for detecting the presence of resistant parasites have long been employed as a surrogate for efficacy trials.

#### In vitro* susceptibility testing is of limited use for monitoring chemoprevention efficacy*

In vitro assays for measuring drug resistance provide a direct measure of parasite response to drugs [6], but have proven to be even more limited in scope and suitability for surveillance than clinical trials. In vitro testing is particularly unreliable for antifolate drugs, especially the sulfas, because the tests are exquisitely sensitive to host folate blood levels, which are affected by diet and vary widely among different individuals [7]. For all these reasons, in vitro tests have not played a significant role in assessing resistance in relation to the currently recommended chemoprevention strategies. Nevertheless, in vitro methods are indispensable for confirming and characterizing newly emerging forms of resistance and for establishing and confirming the molecular mechanisms of resistance [8, 9].

#### Molecular resistance markers can be useful surrogates for efficacy

Elucidation of the molecular basis of in vitro *Plasmodium falciparum* resistance to the antifolates made it possible to define the determinants of in vivo resistance to these drugs, and to develop simple assays for molecular markers of antifolate resistance that can potentially serve as surrogate indicators of drug efficacy. Pyrimethamine and other antifolates such as proguanil (via its metabolite cycloguanil) and trimethoprim target *P. falciparum* dihydrofolate reductase (DHFR), while sulfadoxine and other sulfas target dihydropteroate synthase (DHPS). Resistance to DHFR inhibitors and sulfa drugs in vitro is conferred by single nucleotide polymorphisms (SNPs) in *P. falciparum* DHFR and DHPS, respectively [10–15]. Mutations in both genes tend to occur in a progressive, step-wise fashion, with higher levels of in vitro resistance occurring in the presence of multiple mutations.

Potential molecular markers have been identified for *P. falciparum* resistance to many but not all anti-malarial drugs [16, 17]), including currently used chemoprevention agents such as amodiaquine (SNPs in *pfcrt* and *pfmdr1*), lumefantrine, mefloquine (copy number variation in *pfmdr1*), piperaquine (copy number variation in *plasmepsin2* and SNPs in *pfcrt)*, and the artemisinins (SNPs in *kelch13*). Several of these putative markers are less well validated than those for SP and chloroquine resistance, and in some cases (e.g., lumefantrine), “resistance” markers are associated only with modest differences in susceptibility in vitro but not with clinical measures of resistance or treatment failure.

These resistance markers generally correlate very well with in vitro measures of resistance, but relationships between resistance mutations and chemoprevention outcomes are less straightforward, primarily because intrinsic drug resistance is only one of many factors that affect these outcomes, along with drug quality, intake, absorption, metabolism and clearance; nutritional and other health status indicators; and, especially, naturally acquired immunity to malaria, which can aid in clearing parasites, including drug-resistant parasites [18–22]. Because all these factors vary widely across individuals and populations, validating molecular markers as useful tools for measuring and monitoring drug treatment efficacy and chemoprevention outcomes has been challenging, and no marker or set of markers (haplotype) can reliably predict the outcome of a given drug regimen in an individual person.

Nevertheless, many clinical trials and epidemiological studies have demonstrated strong and consistent associations between the presence of specific mutations and outcomes of interest for both treatment and chemoprevention regimens, particularly those that rely on the antifolate drug sulfadoxine-pyrimethamine (SP). In most settings the presence of *dhps* K540E, which is highly prevalent in East African but scarce in West Africa, reliably signals the presence of four other key mutations, making it possible to use this single marker as a surrogate for the *dhps* “quintuple mutant” that is strongly associated with SP treatment failure [23], a strategy that has been recommended by the WHO for monitoring the efficacy of IPTi with SP [24].

Correlating parasite genetic markers with clinical outcomes can be even more challenging for chemoprevention than it is for treatment efficacy. This is because the relationships between parasite genotypes and efficacy outcomes are comparatively more straightforward in the case of drug treatment of clinical malaria. Drugs are administered and parasites are either cleared or not over the ensuing days. While factors other than resistance affect outcomes for both drug treatment and chemoprevention strategies, in the latter instance, the outcome is less immediate (e.g., birth outcomes following two or more doses of SP administered weeks apart), and the other factors influencing outcome are likely to play a more prominent role in chemoprevention outcomes.

In summary, drug resistance is but one of many factors that determine the efficacy of IPTp, IPTi, SMC and MDA. Clinical trials that measure health outcomes are the gold standard for measuring the efficacy of these chemoprevention strategies. Clinical trials of treatment efficacy cannot be used as a surrogate for chemoprevention efficacy. For antifolates and some other drugs, molecular markers accurately indicate the presence of drug resistant parasites, and are a useful but imperfect means of predicting the efficacy of chemoprevention strategies.

### Impact of chemoprevention on resistance

#### Chemoprevention selects for drug resistant parasites

More than 60 years ago David Clyde showed that the prevalence of antifolate-resistant parasites increased rapidly and dramatically in Tanzanian villages whose residents received weekly pyrimethamine for malaria prophylaxis [25]. Parasitological evidence of resistance was also detected in nearby villages whose residents did not receive chemoprevention, with the highest rates of resistance found in villages closest to those whose residents received pyrimethamine. The molecular basis for this rapid emergence and spread of resistant parasites was demonstrated 45 years later when this field experiment was repeated in a village in Mali, where resistance-conferring mutations in *P. falciparum dhfr* rapidly and dramatically increased in prevalence in the village within just a few weeks of starting all consenting villagers on weekly pyrimethamine [26]. Please note that in this review, “prevalence” of a given mutation or haplotype is defined as the proportion of infected individuals in whom that marker or haplotype is detected, irrespective of whether other alleles or haplotypes (e.g., wild-type) are also present in the infection.

Many subsequent studies have confirmed that community use of SP, whether for treatment or chemoprevention, is often followed by increases in community prevalence of resistance mutations in both *dhfr* and *dhps*. In many of these studies, only very general temporal or ecological trends are reported that are consistent with, but not proof that, various chemoprevention strategies directly select for forms of resistance that affect clinical outcomes. For example, one report described trends of increasing prevalence of molecular markers for antifolate resistant *P. falciparum* in Kenya over a 20-year period when SP was in use, initially for treatment, and subsequently for IPTp [27]. While one novel *dhps* mutation, S436H, more than doubled in prevalence between 2010 and 2017/2018, most of “the usual suspects” of *dhfr* and *dhps* mutations that have been associated with reduced efficacy of SP treatment and chemoprevention were already at near-fixation in 2000, and their prevalence rose only marginally: *dhfr* N51I was prevalent at 90% in 2005, 99% in 2010, and 100% in 2017/2018, and *dhps* A437G was prevalent at 98% in 2000 and 2010 and 100% in 2017/2018. Even if the use of SP for treatment and IPTp was chiefly responsible for these increases, such small changes in prevalence would have minimal impact on SP efficacy. Another molecular survey pooled data from nearly 40,000 samples collected in 38 African countries between 1998 and 2018 [28], and found generally higher prevalences of *dhps* A581G in East compared to West Africa, with extensive heterogeneity including within countries.

While Clyde’s Tanzania study [25] and subsequent observations of the rapid selection of resistance mutations suggest that new forms of resistance can easily emerge locally under drug pressure, genomic epidemiology surveys have found that the most highly resistant forms of resistance to nearly all anti-malarial drugs do not tend to arise de novo wherever drugs are used; rather, parasites with levels of resistance sufficient to cause treatment failure have arisen just a few times, usually in Asia, before spreading to Africa [29–32] (reviewed in [22]). This means that before highly resistant parasites have arrived in an area, even heavy drug selection pressure may not lead to loss of efficacy, as may be the case for antifolate resistance in much of West Africa. However, once highly resistant parasites have been imported and are present even at low prevalence, they can increase in response to drug pressure, as appears to be the case with antifolate resistance in much of East Africa. As more genomic epidemiology studies are undertaken, they are uncovering exceptions to the general rule that clinically relevant forms of resistance tend to emerge in Asia and spread to Africa. It is also possible for new, highly-resistant variants to arise locally on existing genetic backgrounds, as has been reported for *dhps* A581G in East Africa [33].

One important consideration in evaluating the impact of chemoprevention strategies on resistance is that, if the strategy is effective at reducing malaria infections, the number of resistant infections in a population or setting may decrease even while the proportion of infections that are resistant increases as a result of selection pressure exerted by the chemoprevention drugs. Thus when a chemoprevention strategy is highly effective, such as MDA using an ACT, selection favouring resistant parasites may have minimal public health impact if there are so few post-MDA infections that the resistant parasites in a given individual are rarely if ever transmitted. This is why MDA has tended to work best when it is implemented in parallel with rigorous vector control strategies [34].

In contrast, chemoprevention strategies that are less effective at reducing infections, such as IPTp-SP, may be more likely to result in increasing not only the proportion but the number of resistant infections, since they exert their effect less by preventing infections than by reducing parasite densities. Thus, the impact of chemoprevention on resistance depends both on the probability that resistant parasites emerge in an individual infection, and the probability that such resistant infections occur and are successfully transmitted. Mathematical models that incorporate these factors can be helpful in assessing the impact of specific chemoprevention strategies on drug resistance and efficacy [35].

**Fig. 1:**
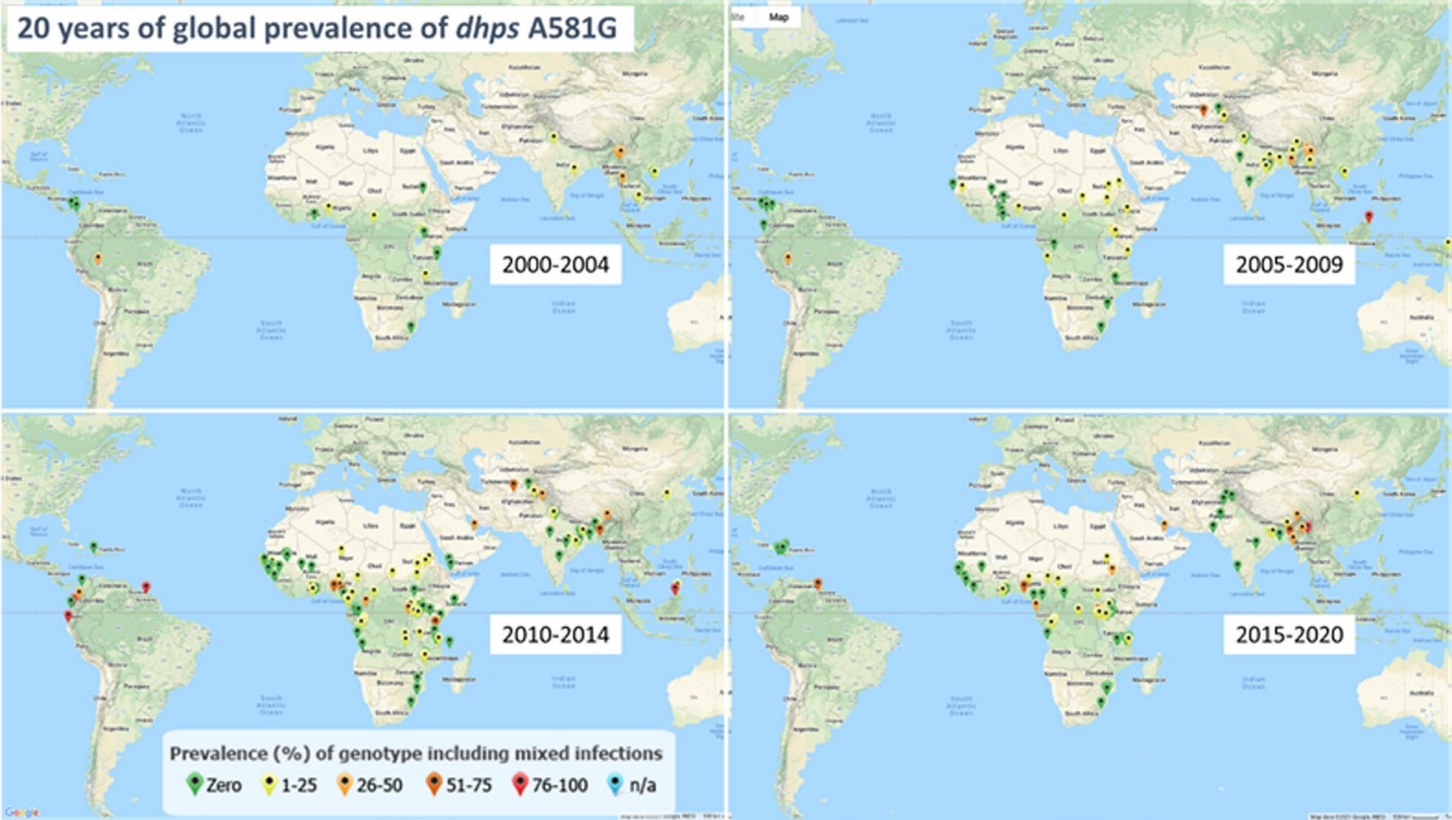
Global map of the prevalence of sulfadoxine-pyrimethamine resistance marker *dihydrofolate reductase* A581G. Data are from published sources and available at http://wwwarn.org/dhfr-dhps-surveyor/#0 (accessed 12 April 2021)

Published data on the prevalence of resistance markers including A581G have been compiled and made available online. For example, Fig. 1 shows global prevalence data for *dhps* A581G between 2000 and 2020. The geotemporal trends for this mutation are consistent with the generally observed pattern of clinically significant resistance mutations being found earlier and at higher prevalence in East as compared to West Africa [22]. The reasons for this pattern are unclear, but plausible potential explanations include: 1) earlier introduction of resistance as a result of more-frequent human migration between Asia, the most common site of origin of highly resistant parasites, and East Africa; 2) more-rapid spread of mutations as a result of higher and more perennial malaria transmission in East Africa; and/or 3) earlier introduction of next-line anti-malarial drugs (first SP, then ACT) in East Africa owing to the earlier emergence of chloroquine resistance there has resulted in earlier and more intense selection pressure favouring parasites resistant to the new drugs in East Africa before these drugs were widely introduced in West Africa.

These global and regional patterns of emergence and spread of drug resistance illustrate a key point about the impact of chemoprevention on resistance: while there are ample examples of malaria chemoprevention strategies being followed by increased drug resistance, it is clear that not every chemoprevention scheme in every setting and population leads to measurable increases in resistance that in turn lead to meaningful loss of drug efficacy in that setting and population. Moreover, it can be difficult to assess the impact of drug use on resistance, and vice-versa, in the many studies that report only prevalences of individual mutations, which by themselves are less reliable predictors of efficacy than full haplotypes. Other issues that commonly cloud interpretation of chemoprevention’s impact on resistance include neglecting to genotype mutations previously believed to be absent from an area, and failure to account for differences in exposure risk among comparator groups in non-randomized observational studies.

### Impact of resistance on chemoprevention efficacy

Antifolate-resistant *P. falciparum* was already well established in Africa by the time IPTp, IPTi, and SMC were implemented there. Based on declining SP treatment efficacy in countries that were early adopters of SP following the rise of chloroquine resistance [23, 36], it was reasonable to expect SP-based chemoprevention strategies to follow a similar pattern. However, IPTp performed well even in settings where antifolate resistance led to SP treatment failure rates of 25% or higher in children [37] (discussed in more detail below).

About eight years after IPTp-SP was recommended by WHO in 1998, reports of increasing SP resistance led to renewed concern that, as one publication asserted, “In northern Tanzania, SP is a failed drug for treatment *and its utility for prophylaxis is doubtful*” (italics added) [38]. This assertion was based on the results of an open label single arm trial of SP efficacy for treating *P. falciparum* in symptomatic children and asymptomatic infants in Korogwe District, about 30 km north of Muheza, Tanzania. The trial had been stopped early owing to an early treatment failure rate of 39% and day 28 failure rate of 82% in the symptomatic children. The authors implicated *dhps* A581G as the culprit in these alarming failure rates, despite multivariate analyses showing that factors associated with treatment failure included young age, high parasite density, and presence of three *dhfr* mutations, but not the presence of *dhps* A581G, which was prevalent at 55%. Notably, the *dhfr* triple mutant had a prevalence of 96% in this study. The findings that this *dhfr* haplotype was at near-fixation in this setting and was nevertheless significantly associated with treatment failure, while *dhps* A581G was not, despite being present in roughly half of the infections, suggests that the lack of association of A581G with treatment failure was real, and not a result of low prevalence or insufficient study power.

This and other studies of SP efficacy for treating clinical malaria in Africa thus raised alarms about the potential impact of antifolate resistance on IPTp and other chemoprevention strategies, but their inconclusive results called for directly examining this question in chemoprevention efficacy trials.

### Intermittent preventive treatment during pregnancy and resistance

#### Impact of IPTp on resistance

As IPTp-SP was being evaluated and implemented in the early 2000s, studies began to examine selection of resistant parasites by ITPp-SP. When *dhfr* and *dhps* mutations were compared in Malawian women from 2003–2006 before they started SP-IPTp and after delivery, the prevalence of the *dhfr/dhps* quintuple mutation increased significantly, from 81% before the intervention to 100% after delivery [39]. Around the same time, studies in other African countries compared marker prevalences in women receiving SP-IPTp and in those not receiving it. The prevalence of *dhfr* mutations was compared in pregnant Ghanaian women at early gestation who had not received IPTp, and in women at delivery, nearly all of whom had received at least one dose of ITPp-SP [40]. Prevalence of the *dhfr* triple mutant was similar between the two groups and did not increase with an increasing number of IPTp-SP doses. Thus, even though the overall prevalence of *dhfr* mutations in the study population doubled between 1998 and 2006 in parallel with the implementation of SP-IPTp, the authors suggested that SP-IPTp might not be responsible for this increase. Similarly, in a study of peripheral and placental samples obtained from pregnant women over a 13-year period in western Kenya, the prevalence of the *dhfr/dhps* quintuple mutant rose contemporaneously with the implementation of IPTp-SP [41]. However, presence of the quintuple mutant was not associated with IPTp-SP use in multivariate analyses, suggesting that other factors were chiefly responsible for its rising prevalence.

In Mozambique, the prevalence of the quintuple mutant was higher in placentas of women receiving IPTp-SP than those receiving a placebo [42]. This association was only significant in women who had received a dose of SP within the 2.5 months before delivery, reflecting the “selection window” [43, 44] during which blood concentrations of sulfadoxine and pyrimethamine remain sufficient to select resistant parasites. In an ITPp-SP study done in Burkina Faso in 2014–2015, *dhfr* and *dhps* triple mutants were more common at delivery than at first antenatal care visit, but the same mutations were even more common in the general population than in pregnant women at either encounter, and recent use of ITPp-SP was not associated with increased prevalence of mutations [45]. In this study, *dhps* K540E was very rare, and *dhfr* I164 and *dhps* A581G and A613S/T were not assessed. Another study in Burkina Faso reported a similar increase in lower-level *dhfr* mutations, but no increase in *dhps* mutations [46].

As *dhps* A581G began to rise in prevalence in Africa, more studies focused on this mutation, which typically occurred together with the other *dhfr* and *dhps* mutations comprising the quintuple mutant to form the so-called sextuple mutant. In a Tanzanian study discussed at length in the next section, *dhps* A581G prevalence was significantly higher in IPTp-SP recipients compared to pregnant women who had not received IPTp [47]. Surprisingly, a survey done ten years later found that A581G was rare or absent in all but one of seven sites in Tanzania [48].

The prevalence of resistance markers before and during IPTp with SP or dihydroartemisinin-piperaquine was compared in clinical efficacy trials conducted in two Ugandan districts, Tororo in 2014–2015, and Busia in 2016–2017 [49]. The *dhfr/dhps* quintuple mutant was already near fixation at both sites, while *dhps* A581G was absent in Tororo and prevalent at only 3% in Busia. Mutations associated with 4-aminoquinoline resistance, *pfmdr* N86Y and Y184F and *pfcrt* K76T, all appeared to be selected in the dihydroartemisinin-piperaquine arms of both trials. The *dhfr/dhps* quintuple mutations were all already prevalent at > 90% and did not increase significantly in the SP arms at either site. The prevalence of *dhfr* I164L remained less than 2% both before and during IPTp-SP in Tororo, but I164L rose in prevalence from 4% to 13.7% in Busia. This is consistent with selection by IPTp-SP at this site, but prevalence of this mutation also rose to 9% in women in the dihydroartemisinin-piperaquine arm who were unexposed to SP, so it is not possible to distinguish between selection by ITPp and community-wide trends in prevalence in Busia over the course of the study. The *dhps* A581G mutation remained absent in Tororo and did not increase in prevalence in either arm in Busia, decreasing from 3% at baseline to 0% in the dihydroartemisinin-piperaquine arm and to 1.9% in the SP arm. The authors speculated that the apparent lack of selection of A581G in Busia was due to its low baseline prevalence. This explanation is unconvincing, in that sharp increases in the prevalence of resistance, and resistance markers, is commonly seen under antifolate drug pressure for other antifolate mutations found at low baseline prevalence [25, 26]. Another study reported apparent selection favouring A581G in Uganda after a single dose of IPTp-SP, but this conclusion was based on very small numbers: A581G was found in two of 52 infected women at the first antenatal visit, compared with two of 12 at the second visit [50].

The *dhps* A581G and A613S/T mutations were reported to be selected by IPTp-SP in another study of antifolate resistance marker prevalence conducted in Ghana in 2015–2017. This cross-sectional study compared marker prevalence in pregnant women at their first antenatal visit and at delivery [51]. At delivery more than 70% of women had received at least two doses of IPTp-SP, so parasites were presumed to have been under selection pressure from SP. Unlike in the contemporaneous Ugandan study that found no increase in prevalence in *dhps* A581G, this West African study saw A581G increase from 9 to 16%. Statistical analyses were not presented, but this increase is not statistically significant (uncorrected *X*^*2*^ = 2.95, P = 0.09). While A613S/T had similar prevalence before and after delivery (15.2% to 17.5%, uncorrected *X*^*2*^ = 0.82, P = 0.37), the authors reported in the abstract that a septuple mutant with both A581G and A613S/T increased significantly from 6.1% at enrolment to 18.2% at delivery (P = 0.03). These results are difficult to compare with those of studies in East Africa, most of which have found that A581G is usually accompanied by K540E. In contrast, in this study A581G was always accompanied by the *dhps* triple mutant and *dhps* S436G, A437G and A613S/T, but never by K540E. Another recent West African survey, of asymptomatically infected pregnant women in Nigeria, similarly found that K540E was absent despite high prevalences of A581G (71%), S436A (55%) and A613S/T (36%) [52], and a survey in Ghana reported similar results [53]. This tendency for K540E to be uncoupled from A581G at some West African sites likely reflects the global patterns of spread of *dhps* haplotypes, with more highly resistant forms commonly found in East Africa having Asian origins, while less resistant homegrown *dhps* haplotypes predominate in West Africa [32].

The single study that provides the most convincing evidence that ITPp-SP does not strongly select *dhps* A581G comes from a well-designed randomized clinical trial done in Malawi in 2011–2014 [54]. Pregnant women were randomized to one of two intervention arms: standard IPTp-SP, or intermittent screening by rapid diagnostic test (RDT), and treatment of RDT-positive infections with dihydroartemisinin-piperaquine. No differences were found in the prevalence of *dhps* A581G in either the peripheral or placental blood among women in the IPTp group who had been exposed to SP, compared to women randomized to the screen-and-treat group who were not exposed to SP.

In summary, IPTp-SP appears to select for antifolate resistance mutations associated with low to moderate increases in drug resistance, but there is no convincing evidence of selection favouring the key mutations—especially *dhps* A581G—associated with higher level antifolate resistance and loss of ITPp-SP efficacy.

#### Impact of resistance on IPTp efficacy

The most recent WHO Guidelines for Malaria continue to recommend IPTp-SP for women living in areas of moderate-to-high transmission in Africa, including in areas with > 90% prevalence of the *dhfr/dhps* quintuple mutant [55]. The guidelines note that where infections with the quintuple mutant plus either *dhfr* I164L or *dhps* A581G are prevalent, “…the efficacy of IPTp-SP may be compromised. It is unclear by how much.” The following discussion considers whether currently available evidence can add clarity on this topic.

Many studies of widely varying quality have assessed the impact of SP resistance on IPTp efficacy. An influential systematic review and meta-analysis published in 2007 pooled data from seven clinical trials of IPTp-SP in relation to SP efficacy for treating symptomatic malaria in young children at or near the same times and locations of the IPTp trials [37]. The authors concluded that even in areas where SP had lost treatment efficacy in children (day 14 treatment failure rates of 19–26%), IPTp-SP continued to provide important health benefits to HIV-negative semi-immune pregnant women and their infants. Moreover, they found no evidence of a substantial loss of IPTp efficacy as SP treatment failure rose from 3 to 39% across sites. In women living with HIV, a group in which IPTp benefit is reduced, IPTp efficacy did decline with rising treatment failure.

The discordance between IPTp-SP benefit in HIV-uninfected pregnant women and SP treatment efficacy in children was attributed mainly to greater levels of acquired immunity in pregnant women. This systematic review did not directly address drug resistance as distinct from treatment failure, nor did it examine relationships between *dhfr* and *dhps* mutations and IPTp outcomes. Nevertheless, policymakers were reassured by the persistent benefit of IPTp-SP in the face of high rates of SP treatment failure in children, and the WHO recommended adopting IPTp-SP in Africa even where the prevalence of parasitological failure at Day 14 after SP treatment among children was as high as 50%, or even higher in areas where IPTp was already implemented [56].

A study done in the same region of Tanzania where earlier studies had found that rising prevalence of *dhps* A581G curtailed the efficacy of both SP treatment of children and IPTp-SP, appeared to support the concern that this mutation boded ill for ITPp-SP [47]. This study, which assessed clinical, parasitological, and histopathological outcomes of IPTp, was even more alarming than the report of very high SP treatment failure rates in Tanzanian children [38]. Based on a study in mice showing that resistant parasites grew to unexpectedly high densities when drug treatment eliminated sensitive parasites [57], the authors hypothesized that, with its compromised efficacy, SP might “select resistant parasites and exacerbate infections in the placenta”. SP resistance mutations, placental parasite densities, and placental inflammation were assessed in women enrolled at delivery between 2002 and 2005 who reported having received, or not having received, SP-IPTp. Those who reported receiving IPTp were classified as “recent IPTp” if they had measurable sulfa levels in their blood, and “early IPTp” if sulfa levels were undetectable.

The authors reported that IPTp was associated not only with higher prevalence of *dhps* A581G but with dramatically higher placental parasite density, and, most concerning, with increased placental inflammation. They reasoned that inflammation indicates chronic placental malaria infection; that inflammation should thus be absent in acute placental malaria; and that placental parasite density normally decreases as placental inflammation increases. Based on these expectations and the observation that inflammation was more common in women who received IPTp, they deduced that the high parasite densities could not be attributed to new acute infections and, therefore, must have resulted from the greater presence of resistant parasites carrying *dhps* A581G in the women who received IPTp. In a subsequent publication of data from the same observational study, the authors reported that IPTp did not reduce the odds of placental malaria, increase mean maternal haemoglobin, or increase birthweight, and IPTp was associated with lower cord haemoglobin and increased risk of foetal anaemia [58]. The implications for ITPp-SP seemed ominous.

This dire interpretation, however, depended on the inference that differences in outcomes were the result of IPTp and not other confounding factors in what was essentially a retrospective case–control study nested within a birth cohort. And while most baseline characteristics showed no significant differences between women who did or did not report receiving IPTp, there was one important, highly significant difference: only 29% of women who received no IPTp lived in rural areas, while 68% of women who received IPTp lived in rural villages. The paper did not discuss differences in malaria epidemiology or risk between the rural and urban sites. An earlier paper describing the parent birth cohort study [59] cited an annual entomological inoculation rate (EIR) of around 400 infected bites/person/year for the study area of Muheza District, but that paper in turn cited another paper from a decade earlier that reported heterogeneous transmission in Muheza, with EIRs ranging from 34 to 405 [60].

With the only available data on transmission intensity in the study area being a ten-year-old study that reported a more than tenfold range of EIRs, it is difficult to dismiss the baseline observation that significantly more rural women had received IPTp. A plausible alternative explanation for the higher parasite densities and placental inflammation in women who received IPTp would be that rural women may have been exposed to up to tenfold higher malaria transmission intensity than their peri-urban counterparts, increasing their risk of acute-on-chronic placental infections. Bed net use was also different at baseline: women who reported no IPTp also reported marginally and insignificantly higher use of bed nets (76.5% vs. 64.4%). However, while none of the (more urban) non-IPTp women reported using insecticide-treated nets (ITN), 16% of (more rural) women who had received IPTp reported using ITNs. The complete absence of ITN use among the non-IPTp women is consistent with alternative explanations, including the possibility that the parasitological and histopathological findings attributed to IPTp selection of A581G-carrying resistant parasites were actually a result of baseline differences in malaria risk between women who received IPTp and those who did not.

Subsequent studies failed to replicate the disquieting finding that IPTp-SP led to increased parasite growth in a setting with prevalent *dhfr/dhps* sextuple mutants. In another cohort of pregnant women in Korogwe District, less than 30 km from Muheza, *dhfr* and *dhps* were genotyped in samples from women who had *P. falciparum*-positive RDTs, and pregnancy outcomes were assessed [61]. During the study period of 2008–2010, the prevalence of the sextuple mutant with *dhps* A581G in Korogwe was 44%, slightly lower than in nearby Muheza several years earlier. The presence of the sextuple mutant was associated with substantially lower birthweights. However, in contrast to the Muheza cohort, the presence of the sextuple mutant was not associated with whether or not women had received IPTp-SP or with how many doses they received; peripheral parasite density tended to be lower, not higher, in women with the sextuple mutant; and there was no relationship between early or recent IPTp and the effect of *dhfr/dhps* haplotypes on birth weight. Studies in Mozambique [42] and Malawi [62] similarly failed to support the notion that IPTp was harmful in settings with high levels of antifolate resistance, although *dhps* A581G was rare (but present) in both studies.

Another systematic review was published in 2013 by the same group that conducted the 2007 review of IPTp efficacy. A meta-analysis of data from seven trials, one each in Kenya, Tanzania, Zambia, Burkina Faso, and Mali, and two in Malawi, found higher average birthweights and lower risk of low birthweight in women who had received three or more doses of IPTp-SP, compared with those who received only two doses [63]. This association was consistent across sites where the prevalence of the *dhfr/dhps* quintuple mutant—as indicated by the presence of *dhps* K540E—ranged from 0–96%. The *dhps* A581G mutant was not prevalent at any of the study sites. Based on these relatively encouraging findings, WHO recommended at least three doses of IPTp-SP irrespective of the presence of *dhfr/dhps* quintuple mutants [64].

To recap, as of 2013, two high quality systematic reviews [37, 63] and more recent clinical trials [42] and surveys [62] supported the WHO position that IPTp-SP was beneficial and should be used across a wide range of antifolate resistance and SP treatment efficacy. On the other hand, an open label SP efficacy study in children [38] and two observational studies in pregnant women [47, 58, 61] suggested that the sextuple mutant represented a dangerous threat to IPTp-SP efficacy, and might be causing IPTp to be not only ineffective but harmful in pregnancy. Each of these three studies portending bad news for IPTp had significant limitations in design and interpretation, and all three were conducted in two adjacent districts in Tanzania, limiting their generalizability to other sites in Africa, where the sextuple mutant remained mostly rare or absent.

Aiming to resolve these discrepant results, an observational study followed by a clinical trial in Malawi and a multi-country efficacy trial of IPTp-SP efficacy directly examined the relationship between SP resistance and IPTp outcomes. The effectiveness of IPTp-SP was assessed in 2009–2011 in Malawi, where the prevalence of the sextuple mutant was 8.4% [65]. The presence of A581G was associated with an approximately threefold increase in the occurrence of “patent” infections (both PCR and microscopy positive) in both peripheral and placental blood, and with higher parasite densities. However, A581G was not associated with any of the following: (1) histological evidence of active placental infection; (2) mean haemoglobin; (3) anaemia; (4) severe anaemia; (5) pre-term delivery; or (6) infants born small for gestational age. Furthermore, women infected with parasites carrying *dhps* A581G gave birth to infants with slightly higher birthweights and had a nearly twofold lower incidence of low birthweight, although these trends did not achieve statistical significance. And, the finding of higher parasite densities in A581G-carrying infections disappeared when the analysis was limited to women with “patent” infections, i.e., when infections that were PCR-positive but microscopy-negative were excluded from the analysis.

Some methodological issues cloud the interpretation of these results. For example, even though more than 90% of both patent (PCR and microscopy-positive) and “subpatent” (PCR-positive, microscopy-negative) infections were successfully genotyped, the prevalence of A581G was reported to be tenfold lower in subpatent infections, a surprising finding that is not explained by the authors. Further muddying the picture, most of the data are presented as pooled results from two study sites, one rural and one urban, even though the prevalence of A581G was more than twice as high at the rural site. Different microscopy staining and reading protocols were used at the two sites, with more rigorous standards at the urban site in Blantyre, and no quality control procedures were described, raising the possibility that rural–urban differences in both malaria epidemiology and the quality of microscopic diagnosis could account for some of the study findings. As with the Tanzanian study described above [47], it is possible that higher parasite densities attributed to resistant parasites were actually a reflection of higher transmission intensity or other epidemiological differences at the rural site where more A581G-carrying infections were found.

A subsequent trial, also done in Malawi by the same group, randomized pregnant women at three sites in 2011–2014 either to receive standard IPTp-SP or intermittent screening with RDTs and treatment of RDT-positive infections with dihydroartemisinin-piperaquine [54]. By the time of this study, the *dhfr/dhps* quintuple mutant was at near-fixation, and the *dhfr* I164L mutation was absent, while *dhps* A581G had a prevalence of 4% in infections found at enrolment in the IPTp-SP group, and 6% in placental infections at delivery. The presence of A581G in placental infections was associated with a significant decrease in gestational age and lower birthweights, but not with parasite placental density, placental inflammation, maternal haemoglobin level, or weight-for-age Z score. Overall, the timing of SP exposure had no impact on birth outcomes, but in the small group of women who had placental infections with A581G, more recent SP exposure was associated with significantly longer pregnancies and higher birthweights. However, a sensitivity analysis showed that this result was driven by a single premature birth of a very small infant to a woman who had received only a single dose of SP; when this outlier was accounted for, the association between recent SP and birth outcomes among women with A581G was not significant.

Taken together, the results of these two studies in Malawi confirmed a partial diminution of IPTp efficacy against A581G-containing placental malaria, but they did not support findings of the studies that had raised the alarm about ITPp-SP causing harm where A581G is prevalent.

None of the studies described so far that promoted the notion that antifolate resistance in the form of *dhps* A581G spelled doom for IPTp-SP in Africa were prospective, controlled trials designed specifically to address this question. In contrast, the relationship between antifolate resistance mutations and efficacy of IPTp-SP was prospectively assessed in a multi-country trial among asymptomatic, microscopy-confirmed *P. falciparum*-infected, HIV-uninfected, pregnant women. Prospective efficacy studies were undertaken between 2009 and 2013 at eight sites in six African countries spanning the continent and a range of prevalences of mutations in *dhfr* and *dhps* [66]. With weekly follow-up, treatment failure was defined as smear-positive *P. falciparum* on or after day 4, with both uncorrected and PCR-corrected efficacy estimates. Resistance genotyping was done using pooled sequencing, so any novel mutations should have been detected.

Study sites were characterized as having low, moderate, or high SP resistance, based on prevalence of *dhps* K540E of < 10%, 10–90% or > 90%, respectively. Defining SP resistance on the basis of this one mutation was reasonable, in that K540E serves as a good surrogate for the *dhfr/dhps* quintuple mutant in settings where *dhps* A581G is absent or rare, as it was in the study sites. A581G, the surrogate for the *dhfr/dhps* sextuple mutant, was absent in the moderate (Zambia) and low (Burkina Faso and two sites in Mali) resistance sites. A581G was found in each of the high resistance sites, with prevalence of less than 0.25% in Uganda, less than 2% in both Malawian sites, and just over 5% in Kenya. At these low levels, it was not possible to assess the relationship between the sextuple mutant and IPTp-SP outcomes in this study.

While SP resistance mutations in this multicentre study did appear to compromise parasite clearance and result in more reinfections as well as a shorter time to reinfection, resistance did not appear to affect birth outcomes. As the authors note, prevalence of resistance mutations was not the only difference across the sites. Although transmission intensity was not measured in this study, malaria transmission has historically been reported to be lower and more sharply seasonal in the West African low-resistance sites, and higher and more year-round in the moderate- and high-transmission sites in East and Southern Africa. This pattern makes it difficult to attribute outcomes solely to resistance. Moreover, lower resistance and transmission might lead us to expect better IPTp outcomes in West Africa, but this same lower transmission may also mean lower natural immunity contributing to poorer outcomes, making it challenging to sort out the relative contributions of these countervailing effects of resistance, exposure risk, and immune protection on IPTp birth outcomes. Finally, women who had been enrolled in the in vivo SP efficacy study were excluded from the birth outcome study. If women who had patent infections early in pregnancy were at higher risk of malaria, their exclusion from the birth outcome study might have reduced the study power to detect SP’s anti-malarial efficacy, lending more weight to non-malaria factors in determining outcomes. Nevertheless, this well-designed study further added to the growing body of evidence that the benefits of IPTp persist in the face of high rates of SP resistance as conferred by *dhfr/dhps* quintuple mutants. The impact of the sextuple mutant (the addition of *dhps* A581G) on IPTp outcomes remained unaddressed by this study, which finished data collection in 2013.

One potential factor contributing to the apparent lack of impact of antifolate resistance mutations on IPTp-SP efficacy is suggested by studies showing that IPTp with either dihydroartemisinin-piperaquine or SP results in comparable pregnancy outcomes despite dihydroartemisinin-piperaquine’s superior efficacy at clearing and preventing malaria infection [67–69]. Studies are underway to assess whether some of SP’s impact on pregnancy outcomes is mediated by non-malaria benefits, e.g., antibacterial activity.

Two additional systematic reviews have attempted to identify a threshold prevalence of *dhps* A581G above which IPTp-SP efficacy is lost. One pooled data from nine IPTp studies (five clinical trials and four observational studies completed at a total of 12 sites) to assess the impact of malaria transmission intensity on IPTp outcomes, as modulated by A581G prevalence [70]. Transmission intensity did not appear to influence the efficacy of IPTp on low birth weight. Data on A581G prevalence collected within two years and 250 miles of the IPTp studies were pooled. Two sites had > 50% prevalence of A581G, and the others had 10% prevalence or less, with four sites having no A581G. Among women who had received two or more doses, IPTp-SP efficacy was preserved at sites with 10% or lower prevalence of A581G. At the two sites with > 50% prevalence, efficacy was diminished but still significant among primigravid and secundigravid women, and absent in multigravid women. The authors concluded that the A581G prevalence threshold above which IPTp-SP should not be used was somewhere between 10–52%.

Another, larger, meta-analysis and systematic review was recently published by the same group that performed the two earlier meta-analyses discussed above, each of which pooled data from just seven studies [37, 63]. This meta-analysis pooled data from 57 studies done in 17 African countries between 1994 and 2014, and confirmed the relationship between prevalence of the quintuple mutant (signalled by *dhps* K540E) and diminished IPTp-SP efficacy [71]. The *dhps* A581G mutation, used as a surrogate for the *dhfr/dhps* sextuple mutant, was present in 16 of the studies, at prevalences ranging from 2.5% to 47%. The pooled analysis thus overcame the sample size limitations of the individual studies that had made it difficult to draw definitive conclusions about the impact of the sextuple mutant in IPTp-SP outcomes. However, the authors’ conclusion that IPTp-SP effectiveness is lost where *dhps* A531G prevalence exceeds 10% is based on just five of the 57 studies included in the meta-analysis.

Three of these five studies had small sample sizes in the reference group and a pooled prevalence of A581G of 21%, and together yielded a relative risk reduction (RRR) of 35%. This means that IPTp-SP retained good effectiveness comparable to that seen in low resistance sites, despite A581G prevalence greater than 20%. Of these three studies, two were from Tanzania and had limitations and discordant results that are discussed at length above [47, 58, 61]. Interpreting results from the third study, from Uganda, is confounded by the unusual finding that both *dhps* A581G and *dhfr* I164L had 36% prevalence, occurring together almost as often as not [72]. The *dhfr* I164L mutation, which has been largely absent or unreported in other studies of IPTp and resistance, is found in parasites with the highest measured pyrimethamine IC50s, approximately tenfold higher than IC50s of parasites carrying the *dhfr* triple mutant. The frequent occurrence of parasites carrying both of these mutations prevents clear attribution of IPTp outcomes in this study to A581G.

Of the five studies that provided the basis for concluding that IPTp-SP efficacy is lost above a 10% prevalence threshold for A581G, the remaining two were from the Democratic Republic of Congo [73] and Uganda [74]. Both of these studies had larger sample sizes and were conducted in areas with a pooled prevalence of A581G of 46%, much higher than that seen in the three smaller studies. Together, these two studies had an RRR of -2% for low birthweight (compared with 35% for the other three studies), signifying a complete loss of IPTp-SP efficacy. This means that among the 57 studies include in the meta-analysis, just two reported that IPTp-SP efficacy was lost where A581G prevalence exceeds 10%. Both studies were also done in areas where the prevalence of K540E was above 90%, making it difficult to attribute the loss of IPTp efficacy to the presence of A581G. Neither of these studies included molecular analyses of *dhfr* or *dhps* mutations—the meta-analysis relied on molecular data collected as close as possible in space and time to the field studies [75–77]. While some of these molecular studies did report prevalence of *dhfr* I164L, the meta-analysis only used data on *dhps* mutations.

In summary, despite some convincing evidence that the presence of *dhps* A581G at least partially compromises the efficacy of IPTp-SP, the worst-case scenario [47] was not borne out by subsequent trials [54, 65, 66]. The evidence supporting a recommendation to withhold ITPp-SP where the prevalence of *dhps* A581G exceeds a threshold of 10% is not strong.

### Intermittent preventive treatment during infancy and resistance

As with IPTp, resistance has been of concern since IPTi was first conceived and evaluated. Most early studies incorporated molecular surveillance to assess the relationships between resistance markers and IPTi-SP efficacy, and to measure the impact of IPTi on the prevalence of resistance markers.

#### Impact of IPTi on resistance

In a 2003–2005 trial of IPTi-SP in aparasitaemic Ghanaian infants, the incidence of *dhfr/dhps* quintuple mutants during two months after the third dose of IPTi was twice as high in the treatment group compared a placebo group [78]. In contrast, the prevalence of *dhfr* triple and *dhfr/dhps* quadruple mutants (*dhfr* triple mutant plus *dhps* A437G in the same infection) remained stable over a one-year period as IPTi-SP was implemented in Mali in 2006–2007, with no differences in marker prevalences between 11 IPTi implementation zones and 11 control zones [79].

Ecological surveys accompanied IPTi-SP evaluations in Tanzania (2004–2007) [80] and Senegal (2006–2008) [81]. Two years after implementation of IPTi-SP, prevalence of the *dhfr* triple mutant in both countries was significantly higher in areas subjected to IPTi compared to nearby control areas. Prevalence of *dhps* A437G was also significantly higher in IPTi areas in Senegal, where *dhps* K540E was absent. While the prevalence of the *dhps* A437G/K540E double mutant was also higher in IPTi areas than in control areas in Tanzania, this difference was not significant.

Follow-up surveys in Senegal in 2009–2010 confirmed that *dhps* K540E remained absent both in zones where IPTi-SP (or IPTc-SP, as seasonal malaria chemoprevention was then called) had been implemented, as well as in control zones. The *dhps* A437G mutation decreased in prevalence in both IPT and control zones between 2009 and 2010. In the IPT zone both A581G and A613S declined from 3 to 0% and from 5 to 0%, respectively, while in the control zone A581G and A613S both rose from 0 to 3% in prevalence during the same two-year period.

In summary, while IPTi-SP has been accompanied by overall increases in the prevalence of some antifolate resistance markers, neither clinical trials of IPTi nor ecological surveys comparing IPTi implementation zones to control areas over time have shown evidence of significant selection of the *dhfr/dhps* haplotypes associated with reduced SP efficacy for treatment or chemoprevention. This conclusion is in agreement with that of an Institute of Medicine expert committee discussed in the next section [82], and with results of two independent modelling studies [83, 84].

#### Impact of resistance on IPTi efficacy

In 2008, the Institute of Medicine (now the National Academy of Medicine) in the USA convened an expert committee to evaluate the evidence concerning IPTi-SP efficacy, including an assessment of the impact of antifolate resistance [82]. The committee undertook a detailed review of published and unpublished data, including new meta-analyses of pooled data from pilot studies of IPTi-SP done at six sites in Tanzania, Ghana, Mozambique, and Gabon. They concluded that: (1) SP treatment efficacy for clinical malaria is not a reliable indicator of IPTi effectiveness; and (2) IPTi-SP retained 20–30% efficacy in the face of 40–80% prevalence of the *dhfr* triple mutant. Among the six sites where IPTi showed measurable efficacy were Ashanti, Ghana, where more than 60% of baseline infections had four or more *dhfr*/*dhps* mutations [85]; Tamale, Ghana, where the *dhfr* triple mutant plus *dhps* A437G (i.e., the quadruple mutant) was found in 44% of infected children aged less than five years [86]; and Manhica, Mozambique, where prevalence of the *dhfr/dhps* quintuple mutant in the placebo arm was 44% [87]. The committee was unable to evaluate the impact of *dhfr* I164L because it was absent or rare at African sites where studies had looked for this mutation. The committee did not consider the role of *dhps* mutations in IPTi efficacy in more detail, owing to the paucity of available data at a time when few studies had examined the role of *dhps* K540E in IPTi efficacy, and the A581G and A613S/T mutations were still rare in Africa.

Subsequent studies found that IPTi-SP efficacy diminished in parallel with rising prevalence of the *dhfr/dhps* quintuple mutant. While the sample sizes were small, all nine genotyped baseline infections had the quintuple mutant in an IPTi trial Korogwe, Tanzania, where four infections (44%) also carried the *dhps* A581G mutation [38]. Although marker prevalence was not reported for infants participating in a trial in Same, Tanzania, a 2001 survey of two sites nearby had found a 60–75% prevalence of the *dhfr* triple mutant and a 43–55% prevalence of the *dhps* double mutant [88]. Neither of these two trials demonstrated significant protective efficacy of IPTi-SP [89].

A pooled analysis of results from seven IPTi trials conducted between 1999 and 2008 found that the duration of protective efficacy was shortened by 50% in the presence of quintuple mutant parasites, from 42 days in Navrongo, Ghana (no *dhfr/dhps* quintuple mutant), to 21 days in Korogwe, Tanzania (89% prevalence of the quintuple mutant) [90]. This meta-analysis also found that protective efficacy in the 35-day period after the 9-month dose of IPTi-SP decreased with an increasing number of resistance markers, although there were not enough data points to determine the effects of specific markers. These data are consistent with a meta-analysis that found that the duration of post-treatment chemoprophylaxis for different artemisinin-based combinations was shorter when the prevalence of markers of resistance to the ACT partner drug was higher [91].

Based on the data available at the time, a 2009 WHO technical consultation recommended that IPTi-SP be implemented only “when parasite resistance to SP in the area is not high”, adding that “Precise cut-offs cannot be defined on the basis of available data.” [24] Just a few months later another technical consultation that included expertise in drug resistance reviewed essentially the same body of evidence and recommended that IPTi-SP not be implemented where prevalence of *dhfr* K540E exceeded 50% [24]. This recommendation was based on just two IPTi-SP trials, one showing 21% protective efficacy in Mozambique where baseline prevalence of *dhfr* K540E was 55%, and one in Tanzania showing no significant efficacy where K540E prevalence was 94%.

A subsequent analysis of molecular marker data collected across Africa from 2005–2011 found that, based on the 50% threshold for K540E prevalence, eight East African countries were classified as unsuitable for SP-IPTi; 14 Central and West African countries were classified as suitable; and seven countries could not be classified owning to a lack of available contemporary data [92]. A cost-effectiveness analysis concluded that IPTi-SP remained cost-effective across a range of SP resistance levels, but the analysis did not consider the high-level resistance conferred by *dhps* K540E, A581G and A613S/T, limiting relevance of the study for areas where these mutations are prevalent [93].

In the last decade, few new studies that inform the impact of resistance on IPTi efficacy have been published. When a cluster randomized trial of IPTi-SP in Tanzania found no survival benefit, the authors speculated that drug resistance was one of many possible factors that could account for this finding, along with operational deficits, decreasing malaria transmission, improving vector control, and better case management [94]. A recent Cochrane review of IPTi noted overall trends of declining IPTi efficacy in parallel with increasing antifolate resistance in Africa, but no new data on SP resistance markers underly this observation, so this meta-analysis does not help in more precisely defining a resistance threshold to guide IPTi implementation decisions [95].

As countries consider implementing IPTi or introducing new drug combinations where IPTi-SP has lost efficacy in the face of resistance, studies directly assessing not only efficacy but duration of protection against both asymptomatic infection and clinical malaria episodes in relation to the prevalence of resistance markers would be of value. As was shown for different ACT treatment regimens [91], the benefits of different chemoprevention regimens may be different in areas with different resistance patterns.

**Fig. 2:**
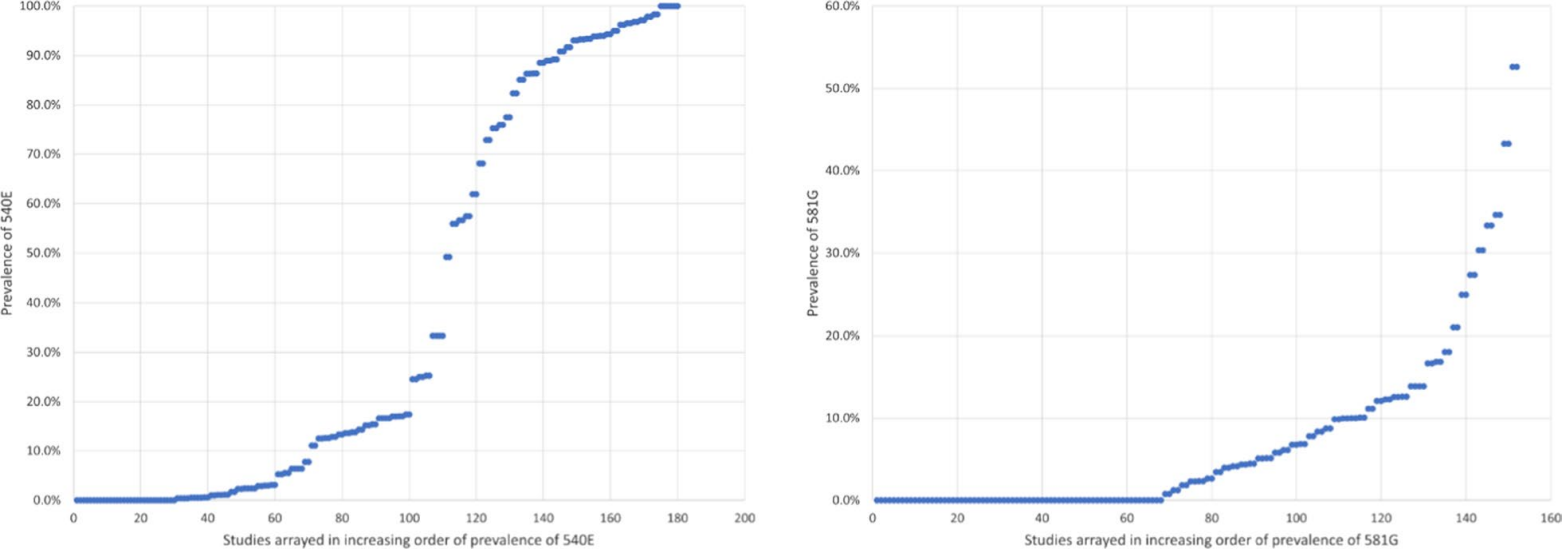
Frequency distributions of prevalence estimates of *dhps* K540E (L) and A581G (R) mutations measured in studies completed in sub-Saharan Africa from 2015–2021. Data were downloaded from http://www.wwarn.org/dhfr-dhps-surveyor and studies completed before 2015 and outside of Africa were excluded. Recent measures of K540E prevalence tend to cluster below 20% and above 50%, while A581G prevalence estimates lack an obvious break point

Given the continued paucity of data on the relationships between SP resistance markers and IPTi efficacy to justify a threshold of resistance above which IPTi should not be implemented or continued, more creative approaches may be needed. For example, Fig. 2 shows the frequency distribution of prevalence measures of *dhps* K540E and A581G for studies completed in sub-Saharan African countries between 2015 and 2021, arranged in increasing order of prevalence. The prevalence of the K540E mutation ranged from 0–100%, with a clear “break point” (sharp change in slope) at around 40% prevalence, providing a natural point for grouping sites with prevalences above and below that point. In contrast, the prevalence of A581G ranged from 0–53%, with a less obvious break point around 15%. When there is a wide range between prevalence levels at which IPTi efficacy persists or is lost, it might be reasonable to choose thresholds based on these break points in the data, to reflect naturally occurring clustering of prevalence levels. This approach might help policy makers avoid difficult decisions when measured prevalences lie very close to the thresholds.

In the case of IPTi efficacy and *dhps* K540E prevalence, based on the observation that IPTi retained 21% efficacy where K540E was prevalent at 55% but not where it was 94%, any threshold between 55 and 94% could have been selected to segregate sites where IPTi-SP might be expected to retain and lose efficacy. The WHO technical consultation recommended a 50% threshold based on the assumption that where prevalence was less than 50%, efficacy should be at least 21%. Based on this new analysis of more recent marker prevalence data as shown in Fig. 2, a case could be made for implementing IPTi where K540E has a prevalence of 40% or lower.

The prevalence of A581G is generally lower, with many studies having 0% prevalence and only two of 152 studies having more than 50% prevalence. Choosing a 10% threshold of A581G for implementing IPTi-SP would be problematic in that eight studies had measured prevalences between 9.9 and 10.1%. Choosing a prevalence threshold of 15% would make it easier for policy makers to segregate sites where IPTi should or should not be used, based on available recent data.

With additional analysis it might be possible to select thresholds based not only on clustering of prevalence estimates, but also geographical clustering. The intent would be to avoid having geographically adjacent areas with prevalence estimates just above and just below a given threshold. Having different IPTi policies in areas that are both geographically close and with similar malaria epidemiology could be confusing to policy makers. Selecting thresholds that would group countries or regions in a logical, understandable fashion could make recommendations easier to understand and follow. For example, choosing a threshold that results in IPTi being recommended in most of francophone West Africa. but not in anglophone East Africa, would be more palatable than one that results in different policies being recommended in coastal and western Kenya, or in northern and southern Tanzania.

These potential new approaches to setting guidelines for chemoprevention when data on resistance and efficacy are limited could be assessed in both field and modelling studies to gauge their utility and feasibility.

In summary, the evidence supporting a recommendation that IPTi-SP not be deployed where prevalence of *dhps* K540E exceeds 50% was thin when an expert group identified this threshold based essentially on just two trials ten years ago, and little new evidence is available to validate this threshold, or to set new criteria to guide IPTi policy (e.g., a prevalence threshold for *dhfr* I164L, *dhps* A581G, and/or *dhps* A613S/T). Efficacy studies of potential new IPTi drug regimens should include assessments of efficacy and duration of protection in relation to resistance markers. Until more evidence is available on the relationship between SP resistance and IPTi-SP efficacy, an alternative approach would be to select thresholds for implementing IPTi based in part on natural clustering of prevalence data in recent studies.

#### Seasonal malaria chemoprevention and resistance

In 2012 WHO recommended another chemoprevention strategy, seasonal malaria chemoprevention (SMC, formerly called Intermittent Preventive Treatment in Children or IPTc). SMC with SP and amodiaquine (SP-AQ) is recommended for children aged less than five years in regions of the West African Sahel with intense seasonal malaria transmission. As recommended by the WHO, SMC consists of a complete treatment course of SP-AQ administered to children aged 3–59 months at monthly intervals, beginning at the start of the transmission season, up to a maximum of four doses during the malaria transmission season. The relatively lower levels of antifolate resistance in West Africa, and the addition of amodiaquine to the regimen, gave rise to optimism that SMC might be less threatened by resistance than IPTp was in East Africa.

#### Impact of SMC on resistance.

In a 2008 trial in Burkina Faso, after three monthly rounds of SMC with SP-AQ the prevalence of infections with *dhfr*/*dhps* quadruple mutants (triple *dhfr* and *dhps* A437G mutants) was comparable in the treatment and placebo arms, with an overall increase over baseline prevalence in both groups [96]. In contrast, a contemporaneous trial in Mali appeared to show SMC selection of low- and mid-level antifolate resistance markers. While the *dhfr/dhps* quintuple mutant (quadruple plus *dhps* K540E) was absent, the prevalence of quadruple mutants was significantly higher in the SP-AQ group than in the placebo group, and prevalence increased from baseline in the SMC group but not in the placebo group [97]. In a trial of SMC with SP plus artesunate in Senegal, the post-intervention prevalence of quadruple mutants was also significantly higher in the intervention arm than the placebo arm, again with an increase in both groups from baseline [98]. Prevalence of resistance markers continued to rise in both groups, and no difference between the intervention and placebo arms was detected after the second year of follow up, possibly as a result of increased SP use in the general population following a change in national first-line treatment policy to SP-AQ [98]. A subsequent comparison of SMC with SP-AQ and dihydroartemisinin-piperaquine in Burkina Faso similarly found evidence of modest selection *dhfr* S108N and C59R and *pfcrt* K76T in the SP-AQ arm of the trial [99].

As noted above, the impact of SMC on resistance is related not only to the proportion of infections that carry resistant parasites, but on the proportion of people who become infected. Modelling studies may be useful in assessing whether SMC’s efficacy at reducing the prevalence of infection mitigates the risks posed by its effect of increasing the prevalence of resistance (defined here as the proportion of infections carrying resistant parasites).

Based on these early studies, it appeared that at least short-term selection of resistance markers may follow SMC implementation. Surveys of health districts that had or had not implemented SMC or IPTi in Senegal found significant selection of the *dhfr* triple mutant, but not for *dhps* mutations [100]. An ecological survey in Ghana that included areas where SMC had and had not been implemented reported similar increases in *dhfr/dhps* quintuple mutants, but this study did not test for the higher-level resistance mutations *dhfr* I164L and *dhps* A581G and A613S/T [101]. Another prospective SMC trial done in Mali in 2014 found that prevalence of the quintuple mutant remained similar and below 5% before and after IPTp-SP was implemented in two districts (this trial also did not assess higher-level SP resistance mutations) [102]. The Mali trial also reported no increases in the prevalence of *pfcrt* or *pfmdr1* polymorphisms associated with diminished AQ susceptibility.

A large observational study of the scale-up of SMC with SP-AQ in seven Central and West African countries measured the prevalence of resistance markers in 2016 and 2018 among 10–30 year-olds to assess the overall trends in resistance markers in communities where under-fives were given SMC [103]. The *dhfr* triple mutant was already prevalent at more than 90% across the sites, and increased yet more; and *dhps* mutations were initially lower and increased proportionally more, with up to fourfold increases in prevalence over time. However, AQ resistance markers in *pfmdr1* and *pfcrt* decreased modestly during the scale-up period. These results are consistent with SMC with SP-AQ selecting for antifolate resistance but not 4-aminoquinoline resistance. However, other plausible reasons for these changes in marker prevalence include reduced CQ use in the region resulting in reduced selection pressure for resistance to 4-aminoquinolines, and other sources of selection pressure favouring antifolate resistance by the use of SP or other antifolates such as trimethoprim-sulfamethoxazole (co-trimoxazole) for antibacterial treatment or chemoprevention. Notably, the fold-increases in the prevalence of *dhps* markers as well as various *dhfr-dhps* haplotypes associated with intermediate to high antifolate resistance were all lower (in many cases, 2–threefold lower) in the under-fives than in 10–30 year-olds, despite the younger group being subjected to direct selection for antifolates under SMC. This marked age difference further clouds the interpretation that SMC was solely responsible for the rise in antifolate markers over the study period.

In summary, while some prospective trials and ecological studies of SMC with SP-AQ in West Africa have reported increased prevalence of the *dhfr/dhps* quadruple and quintuple mutants, other studies found no such evidence of selection. No evidence has been reported of SMC being followed by increased prevalence of the higher-level resistance mutations that most severely impair SP efficacy, nor does SMC appear to select for parasites carrying mutations associated with diminished AQ susceptibility.

#### Impact of resistance on SMC efficacy.

While the *dhfr/dhps* quadruple mutant was already prevalent in West Africa as SMC was being tested and implemented, *dhps* K540E was still rare in the region [104]. SMC efficacy using SP combined with either amodiaquine or artesunate ranged from 70–87% at sites in Senegal, Mali, and Burkina Faso with baseline prevalences of 32–58% of the *dhfr* triple mutant and 22–29% for *dhps* A437G [96–98], suggesting that SMC benefit persists in the face of moderate levels of the quadruple mutant. A meta-analysis of SMC trials was conducted [105], but because baseline prevalence of resistance markers prior to implementation was generally not reported, marker prevalence could not be associated with efficacy, nor could selection be measured. Putative molecular markers for amodiaquine resistance, including mutations in *pfcrt* and *pfmdr1*, have generally not proven reliable predictors of SMC efficacy. For example, a clinical trial of SP, AQ and SP-AQ for treatment of clinical malaria in Cameroon found that prevalence of *pfcrt* and *pfmdr1* mutations thought to be associated with reduced susceptibility to AQ was higher at sites where AQ and SP-AQ treatment failures were lower [106].

In summary, unless and until high-level resistance mutations become more prevalent in areas where SMC is used, it will not be possible to draw conclusions about the impact of resistance on SMC efficacy.

### Mass drug administration and resistance

Mass drug administration (MDA) refers to mass drug treatment of an entire population, irrespective of the presence of symptoms and without individual testing for malaria [34, 107]. During the last century MDA schemes often led to declines in malaria rates, but gains were usually temporary [108]. Exceptions to this pattern include instances of MDA being deployed in combination with aggressive vector control and rigorous surveillance in low-transmission areas, and in geographically conscribed areas, such as islands [34, 109]. MDA was blamed for driving drug resistance, most notably after introduction of anti-malarial drugs in table salt in the 1950s [110], and the WHO stopped recommending it. However, in response to the renewed call for malaria eradication and the emergence of artemisinin resistance, MDA has been re-examined [107, 109, 111]. Trials and implementation projects have been undertaken both in low burden settings slated for elimination such as the Greater Mekong Subregion [112] as well as in Africa [113]. These more recent experiences with MDA have provided the opportunity to gain a better understanding of the impact of MDA on the emergence and spread of resistance, and the impact of drug resistance on MDA efficacy.

#### Impact of MDA on resistance

In MDA, every consenting member of a malaria-exposed population is administered curative doses of anti-malarial drugs, irrespective of infection status. This is often repeated at intervals, e.g., two monthly cycles repeated annually for two years. It would seem obvious that such a massive drug exposure would exert powerful selection pressure favouring resistant parasites—and, indeed, MDA has been indicted for hastening resistance throughout the history of malaria control. Malaria icon Walther Wernsdorfer (who literally wrote the book on malaria) asserted 40 years ago that “Mass drug administration in its various forms, and insufficient treatment are obviously the most important motors of selection.” [114] While this is an oft-repeated notion, the evidence is less clear cut.

Theoretical arguments have been made that MDA prevents rather than fosters resistance, based on calculating probabilities of emergence and spread of resistance in relation to parasite density [115]. This prediction appears to be supported by recent well-executed MDA schemes in low-transmission elimination zones with highly efficacious drugs that found no evidence of selection for drug resistant parasites. For example, mutations in *P. falciparum kelch13* associated with artemisinin resistance were already prevalent when MDA with dihydroartemisinin-piperaquine and low-dose primaquine was evaluated in eastern Myanmar, where a piperaquine resistance marker (multiple copies of the *P. falciparum* genes *plasmepsin2/3*, or *pfpm2/3*) was absent at baseline. There was no evidence of selection of resistance by MDA: after MDA, the piperaquine resistance marker was still absent, and *kelch13* mutations had decreased in prevalence from 86 to 57% [116].

A cluster-randomized trial of MDA with dihydroartemisinin-piperaquine included Southeast Asian sites with varying levels of resistance. MDA was randomly either initiated or delayed in 16 villages with about 500 residents each [117]. A highly resistant parasite lineage with both the *kelch13* artemisinin-resistance mutation C580Y and the piperaquine resistance marker, multiple copies of *pfpm2/3*, was absent at baseline in Myanmar and Lao PDR, but present in Vietnam and Cambodia at prevalences of 4% and 63% of genotyped infections, respectively. Only 14 of the 258 individuals who were infected with *P. falciparum* at baseline and completed three rounds of MDA were persistently infected a month later, 13 in Vietnam and one in Cambodia. Only the single persistent infection in Cambodia carried the highly resistant haplotype.

In Mozambique, where malaria transmission and parasite densities are much higher than in Southeast Asia, the prevalence of resistance markers was compared before and after two annual cycles of two monthly rounds of MDA with dihydroartemisinin-piperaquine [118]. No evidence of selection was found for markers of resistance to artemisinins (*k13*) or piperaquine (*pfpm2* and *pfcrt*).

Modelling studies have both supported and undermined the notion that MDA is a potent force driving resistance. One study concluded that the “windows of selection” for drugs used in chemoprevention were longer than estimated based on clinical data, leading the authors to assert that MDA and other chemoprevention strategies using full treatment regimens “will be far more potent drivers of resistance than previously thought” [119]. However, another modelling study that also incorporated pharmacodynamic properties as well as resistance mechanisms of MDA drugs came to different conclusions. This study found that while MDA using drugs to which parasites can become highly resistant with a single mutation, such as atovaquone, would result in high levels of resistance even after a single round, MDA with artemisinin-based combinations would retain efficacy because of the lower grade of resistance generated by more complex and therefore less frequently occurring genetic mechanisms [120]. The latter model appears to align better with the results of recent MDA experiences with ACT in both low and high malaria transmission settings.

In summary, there is no evidence that MDA in the modern era using highly effective artemisinin-based combination results in increased drug resistance, although studies addressing this topic are limited.

#### Impact of resistance on MDA efficacy.

In early experiences with MDA using sub-curative drug regimens, MDA quickly selected for resistance, which in turn compromised efficacy [25, 34]. However, in more recent MDA schemes in Southeast Asia, the high efficacy of ACT has been preserved, even in areas with more than 60% prevalence of artemisinin resistance, and efficacy has been stable across sites with low and high rates of resistance to both artemisinins and ACT partner drugs [112, 117]. MDA with ACT has been less efficacious in Africa, not because of drug resistance but because of epidemiological and parasitological factors that differ from low-transmission areas slated for elimination. For example, MDA has either failed or been followed by rebounding malaria incidence when it has been attempted in limited areas adjacent to non-MDA areas that serve as a source for rapid re-introduction of malaria to the populations subjected to MDA [34, 109]. Even in lower transmission settings, MDA’s effects are short-lived if it is applied with less-than-ideal rigor in the absence of effective vector control methods [121]. The near-complete absence of clinically relevant levels of resistance to ACT drugs in Africa precludes any assessment of the impact of resistance on MDA efficacy there.

In summary, in the past drug resistance diminished the efficacy of MDA when drugs were used in sub-curative formulations and dosing regimens (e.g., single drugs used at doses that fail to clear infection). However, in the twenty-first century, MDA with highly effective combination drugs has proven efficacious even in the face of high levels of resistance. Nevertheless, policy makers continue to express worries about MDA promoting resistance [122].

### Other potential uses of chemoprevention and resistance

While other potential uses of chemoprevention for malaria control and elimination are not presently recommended by the WHO, evidence from evaluations of new chemoprevention strategies can shed light on the relationships between drug resistance and the WHO-recommended strategies reviewed here. For example, several studies have explored the benefits of preventive drug treatment for malaria among school-age children in East and Southern Africa, where malaria transmission tends to be more perennial than in the West African countries where SMC has been tested and implemented.

A recent systematic review and meta-analysis of preventive treatment among school-age children in Africa that pooled data from 13 studies [123] noted that “…the only study to measure directly the effect of school-based treatment on drug resistance showed that recent treatment with dihydroartemisinin–piperaquine was associated with higher prevalence of molecular markers of drug resistance.” The study in question, from Uganda [124], measured the proportion of *P. falciparum* infections carrying only the “pure mutant” forms of known resistance markers in relation to the time of the most recent dose of dihydroartemisinin–piperaquine given as monthly chemoprevention to school-age children. This analysis thus combined mixed infections (containing both resistant mutant parasites and sensitive wild-type parasites) with pure wild-type infections in the reference (ostensibly non-resistant) group. This analytical approach limited the “resistant” outcome to those infections in which only “pure mutant” forms were detected. For the purpose of assessing selection of resistance and risk of treatment failure, arguably the more appropriate analysis would have been to compare the proportion of infections containing any resistant parasites, whether or not wild-type parasites were also present in the infection. This is because it is the presence of resistant parasites (irrespective of the presence or absence of sensitive parasites) that signals the risk of treatment failure—the additional presence of wild-type sensitive parasites should have no effect on whether or not the resistant parasites are cleared by drug treatment.

**Fig. 3:**
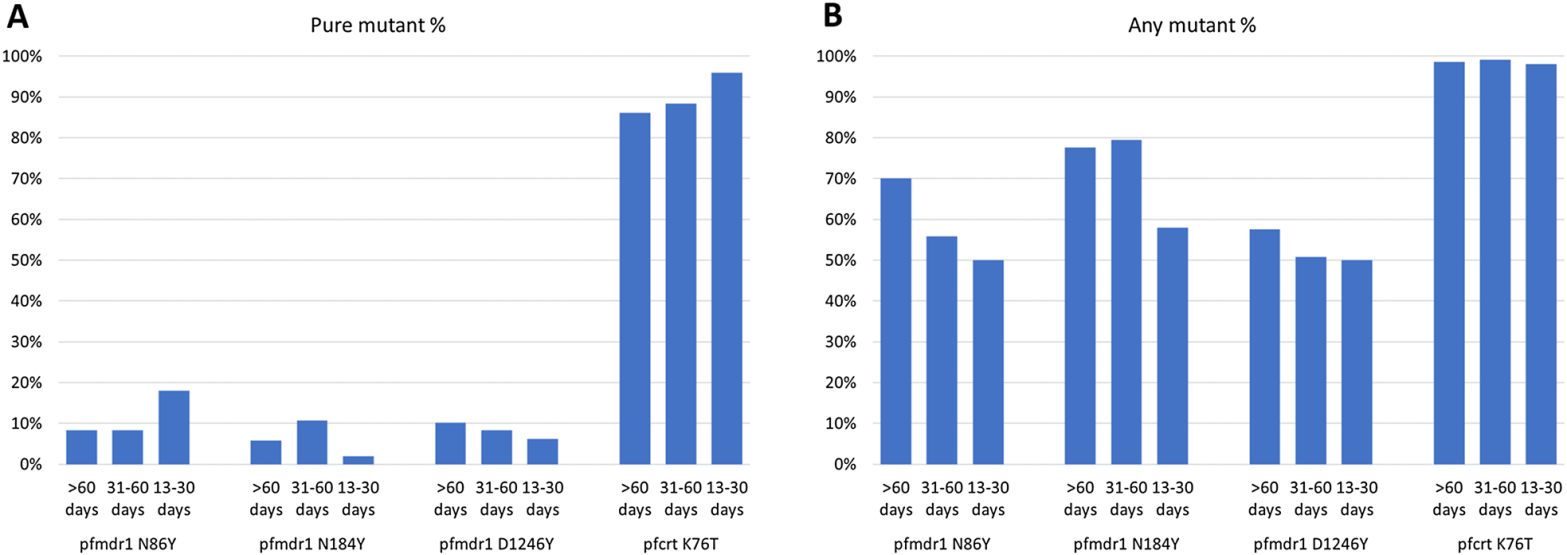
Re-analysis of data purportedly showing selection of resistance markers by monthly seasonal malaria chemoprevention in school-age Ugandan children. For each resistance marker, the three bars represent proportion of infections containing mutant genotypes at increasingly distant times from last drug treatment with Dihydroartemisinin-piperaquine. Panel A shows the original analysis, depicted here in graph form, and showing apparent selection of “pure mutant” genotypes of *pfmdr1* N86Y and *pfcrt* K76T based on their increasing in prevalence after drug treatment. Panel B depicts a re-analysis of the same data showing no evidence of positive selection for mutant genotypes when all infections containing the mutation in question are considered to have resistant parasites. Data from Nankabirwa et al*.* Antimicrob Agents Chemother 2016, 60:5649–54

As shown in Fig. 3, when the data in the Uganda paper [124] are re-analysed using this approach of assessing the presence or absence of resistant parasite genotypes (irrespective of presence of wild-type genotypes), there is no suggestion of increased prevalence of any resistance markers in infections occurring further in time from dihydroartemisinin-piperaquine administration. In fact, one of the resistance markers, *pfmdr1* N86Y, appears to be significantly less prevalent in infections that occurred sooner after drug treatment, consistent with selection favouring wild-type parasites. The other marker that had appeared in the original analysis to be selected by chemoprevention in this setting, *pfcrt* K76T, was prevalent at near-fixation levels in all infections, irrespective of temporal proximity to drug treatment, as shown in Fig. 3.

In summary, the evidence that malaria chemoprevention in school-age children increases drug resistance does not stand up to careful scrutiny. This example illustrates the importance of rigorous study design and analysis in assessing the relationships between drug resistance and malaria chemoprevention strategies and lends further support to the idea that selection of clinically relevant forms of resistance by chemoprevention is not inevitable.

### Potential approaches to manage and mitigate the risk of resistance

The history of antimicrobial use is rife with examples of drugs being used in inappropriate ways that hasten the emergence and spread of resistance, such as overprescribing antibacterial drugs for viral illnesses, or adding antibiotics to livestock feed to enhance animal growth. In the case of malaria, it is hard to dispute the inadvisability of practices like adding anti-malarial drugs to table salt [110] and the unfettered sale and use of drugs of questionable quality in the private sector [125]. Concerns about resistance can trigger policymakers to resist new or expanded uses of valuable drugs. While this protective urge is understandable, and can lead to useful initiatives such as expanding diagnostic capacity to reduce empiric malaria treatment for all fever cases, it comes with a risk of restricting access to beneficial drugs that could be deployed in ways that do not appreciably shorten their useful lifespans. Understanding of resistance mechanisms may offer potential approaches for finding the optimal balance between treating and preventing malaria and preserving drug efficacy.

#### Can countervailing resistance mechanisms be exploited to preserve efficacy?

The WHO and others have recommended that the risk of chemoprevention hastening the demise of treatment drugs should be mitigated by using different drugs for chemoprevention and first-line treatment. IPTp, IPTi and SMC programmes generally follow this recommendation, as SP and SP-AQ are not recommended first-line treatments in countries where these strategies are deployed. Recent MDA programs have been less compliant with this advice, in that MDA with ACT has been used in areas where ACT is also the first-line malaria treatment. This means that the same class of drug—the artemisinins—are subjected to potential selection pressure for resistance in both treatment and chemoprevention regimens, in the same areas if not in the same populations. ACT is likely to remain the first choice for MDA until other equally highly efficacious and well-tolerated regimens are available.

In the meantime, one approach for reducing the potential for MDA to select forms of resistance that impair ACT efficacy is to use different regimens for MDA and first-line treatment, with ACT partner drugs that have antagonistic resistance mechanisms. For example, resistance to mefloquine has been associated with increased copy number of the *pfmdr1* gene [126–128] and piperaquine resistance is associated with increased copy number of the *pfpm2* and *pfpm3* genes [129, 130]. Parasites with increased copy numbers of *pfpm2/3* signalling piperaquine resistance usually occur together with the wild-type single-copy *pfmdr1* associated with mefloquine sensitivity. These antagonistic resistance mechanisms could potentially be exploited to preserve efficacy by deploying artemisinin-based combinations with countervailing resistance selection pressure, e.g., using dihydroartemisinin-piperaquine for MDA and artesunate-mefloquine or artemether-lumefantrine for treatment. A recent trial of ITPp with mefloquine reported apparent selection against the *pfmdr1* N86Y mutation that is associated with chloroquine resistance, raising the possibility that IPTp-mefloquine could drive selection of mefloquine-resistant but chloroquine-sensitive parasites [131].

The two anti-malarial drugs for which counter-resistance is best documented, chloroquine and mefloquine, have recovered efficacy after being withdrawn in some areas and are being evaluated for reintroduction into use. When chloroquine was withdrawn and replaced with SP as the first-line drug in Malawi, chloroquine resistance disappeared over a period of about eight years [132]. Chloroquine was shown to be highly efficacious once again for malaria treatment [133], and weekly and intermittent chloroquine chemoprevention had similar efficacy to IPTp-SP in pregnant women [134]. Chloroquine resistance also declined dramatically after chloroquine was no longer recommended in Tanzania [135] and Zambia [136]. Similarly, after six years as first-line treatment in Thailand, mefloquine efficacy declined from 98% to up to 50% [137]. When dihydroartemisinin-piperaquine was used in the region, mefloquine efficacy recovered, and it is now being studied in the region as a component of a triple ACT [138].

Whether recovery of efficacy results from counter-resistance favouring drugs lost to resistance, or simply resurgence of sensitive parasites in the absence of drug pressure [139], rotating or alternating anti-malarial drugs could be a useful approach for managing resistance. Alternatively, drugs with countervailing resistance profiles could be deployed in parallel: a strategy of “multiple first line therapies” has been proposed to preserve efficacy of treatment drugs [140], and the rationale for “triple therapy” ACT includes the possibility of using drugs with antagonistic resistance profiles [138, 141].

Triple therapy in the form of dihydroartemisinin combined with piperaquine and mefloquine has been proposed as a way to protect ACT partner drug efficacy [142] and is being evaluated for malaria treatment in western Cambodia [138]. Where ACT efficacy is severely compromised triple drug therapy offers a valuable option for malaria treatment, but the added expense and safety considerations make triple therapy less viable for chemoprevention strategies. A recent systematic review of mefloquine for preventing malaria in pregnancy found that while it had superior efficacy to ITPp-SP, high rates of mefloquine-related adverse events limit its potential effectiveness [143]. Other proposed approaches for mitigating or overcoming the impact of resistance on chemoprevention include using antibacterial drugs that have modest anti-malarial efficacy and are thought to be refractory to resistance, such as azithromycin [144, 145], doxycycline [146], or trimethoprim-sulfamethoxazole [147]; increasing the dosage or changing the dosing interval to protect against resistant parasites [148]; and adopting screen-and-treat instead of intermittent treatment [54, 67, 149–151]. None of these approaches has gained acceptance as a viable alternative to IPTp-SP.

Another potential approach for deterring resistance is matching pharmacokinetic properties of drugs used in combination, so that longer-acting partner drugs are not left “unprotected” by persisting at levels that select for resistance after the shorter-acting partner drug has been eliminated [152–154]. Matching half-lives and elimination curves is an attractive approach that should ideally be incorporated into the design of future anti-malarial drug combinations. In the meantime, with the limited number of effective drugs currently available, most drug combinations in use now, and all artemisinin-based combinations, include partner drugs with grossly mis-matched pharmacokinetic profiles. Compared to artemisinin-based combinations, which all pair longer-acting partners with extremely rapidly cleared artemisinins, SP and SP-AQ are reasonably well-matched combinations.

Each of these approaches to mitigating and deterring resistance comes with significant challenges. In discussions about multiple first-line therapies, National Malaria Control Programme managers have explained to researchers and modellers that implementing changes in first-line malaria treatment drugs is not simply a matter of issuing recommendations—doing so effectively requires major investment of resources, time, and effort in training health providers, educating the public, and establishing new procurement and distribution systems. With mathematical models yielding divergent predictions about the benefits of multiple first-line therapies [155], policy makers remained understandably sceptical about this approach.

Proposed chemoprevention strategies that rely on drugs with adverse effects that are tolerable when treating ill patients (e.g., doxycycline, mefloquine) may not be acceptable to the healthy people who are the target population for chemoprevention strategies. Increasing drug dosages to overcome resistance likewise increases safety concerns, especially for use in infants, children, and pregnant women. Screen-and-treat strategies are appealingly efficient, in that they avoid treating uninfected individuals, but they also miss the large reservoir of sub-patent infections and miss out on the post-treatment prophylaxis benefit for people infected shortly after treatment that accounts for much of the benefit of IPT and SMC.

In summary, standardized protocols for measuring and monitoring chemoprevention efficacy are needed. With imperfect evidence, practical considerations such as known prevalence patterns can help guide recommendations on when and where to deploy chemoprevention strategies. Using different drugs for chemoprevention and treatment and combining drugs with countervailing resistance mechanisms may help to preserve efficacy. The best approach for mitigating and managing drug resistance to protect the efficacy of chemoprevention strategies is to ensure that there is a pipeline of safe and effective new malaria drugs, ideally with diverse mechanisms of action and resistance, to replace those lost to resistance.

### Summary and final perspectives

The evidence reviewed here about the relationships between drug resistance and malaria chemoprevention strategies comes from a patchwork of studies of diverse designs and varying quality that sometimes yield conflicting results. Studying the relationships between resistance and efficacy is only possible where there is both a high enough prevalence of resistance and high enough level of efficacy to measure associations with adequate statistical power. The heterogeneous settings, populations, and malaria epidemiologies where chemoprevention strategies are tested and used limit the generalizability of individual studies.

Meta-analyses of pooled data have been helpful in guiding policy recommendations, but even large meta-analyses are limited by the small numbers of well-designed studies in which data on resistance were directly collected. This can mean that seemingly robust analyses that draw conclusions based on pooled data from dozens of studies may in fact base those conclusions as few as one or two studies. Most of the meta-analyses reviewed here also pooled molecular marker data from separate surveys that were done as close as possible in time and space to chemoprevention trials. Conclusions thus rely on the suspect assumption that the prevalence of molecular markers is stable across time, space, and populations.

These limitations in the quality and comparability of the available data mean that it is much easier to draw conclusions about what is not known than to develop clear evidence-based guidance based on what we do know. For this reason, many of the key findings summarized in Table 2 are conclusions that the evidence justifying various recommendations is insufficient or weak. Ultimately, health policy makers must make decisions in the face of substantial uncertainty. For example, the WHO has recommended specific molecular marker prevalence thresholds above which certain chemoprevention strategies should not be implemented. The available evidence may support only wide ranges—if the data tell us that a given strategy is likely to retain efficacy if the prevalence of a given marker is somewhere between 10–50%, do we recommend a threshold of 10%, or 50%, or something in between?

**Table 2:** Summary of key findings

Measuring and monitoring resistance	Drug resistance is but one of many factors that determine the efficacy of IPTp, IPTi, SMC and MDA Clinical trials that measure health outcomes are the gold standard for measuring chemoprevention efficacy Drug treatment efficacy is not a reliable surrogate for chemoprevention efficacy Molecular markers accurately indicate the presence of drug resistant parasites, and can serve as useful but imperfect means of predicting chemoprevention efficacy Specific resistance markers must be validated independently as predictors of efficacy for each different chemoprevention regimen
Impact of IPTp on resistance	IPTp-SP appears to select for antifolate resistance mutations associated with low to moderate increases in drug resistance, but there is no convincing evidence of selection favouring the key mutations associated with higher level antifolate resistance and loss of ITPp-SP efficacy
Impact of resistance on IPTp	Despite some evidence that high level antifolate resistance at least partially compromises IPTp-SP efficacy, a worst-case scenario of harmful effects in the presence of SP resistance was not borne out by subsequent studies The evidence supporting a recommendation to withhold ITPp-SP where the prevalence of *dhps* A581G exceeds a threshold of 10% is not strong
Impact of IPTi on resistance	While IPTi-SP has been accompanied by overall increases in the prevalence of some antifolate resistance markers, there is little evidence of significant selection of the forms of resistance known to compromise SP efficacy for treatment or chemoprevention
Impact of resistance on IPTi	The evidence supporting a recommendation that IPTi-SP should not be deployed where prevalence of *dhps* K540E exceeds 50% remains limited
Impact of SMC on resistance	While some studies have reported that SMC is followed by increased prevalence of resistance markers, other studies found no such evidence of selection There is no evidence that SMC results in increased prevalence of the higher-level resistance mutations that most severely impair SP efficacy, nor does SMC appear to select for parasites carrying mutations associated with amodiaquine resistance
Impact of resistance on SMC	Unless and until high-level resistance mutations become more prevalent in areas where SMC is used, it will not be possible to draw conclusions about the impact of resistance on SMC efficacy
Impact of MDA on resistance	There is no evidence that MDA in the modern era using highly effective ACTs results in increased drug resistance
Impact of resistance on MDA	In the past, drug resistance has diminished the efficacy of MDA when drugs have been used in sub-curative formulations and dosing regimens However, in the twenty-first century, MDA with highly effective combination drugs has proven efficacious even in the face of high levels of resistance
Other chemoprevention strategies	Evidence that seasonal malaria chemoprevention in school-age children increases drug resistance does not stand up to careful scrutiny Selection of clinically relevant forms of resistance by chemoprevention is not inevitable
Managing and mitigating resistance	Standardized protocols for measuring and monitoring chemoprevention efficacy are needed With imperfect evidence, practical considerations can help guide recommendations on when and where to deploy chemoprevention strategies Using different drugs for chemoprevention and treatment and combining drugs with countervailing resistance mechanisms may help to preserve efficacy The best approach for mitigating and managing drug resistance to protect the efficacy of chemoprevention strategies is to ensure a pipeline of safe and effective new malaria drugs with diverse mechanisms of action and resistance

Recommendations may need to be tempered to offer broader guidelines than precise prevalence thresholds for resistance markers. For example, guidelines may include statements along the lines of: “IPTx has been shown to be efficacious in settings with a prevalence of [resistance marker] up to XX% but not in a setting with a [resistance marker] of YY%. The relationship between efficacy and mutation frequencies between XX% and YY% remains unknown. Resistance may not have been the only factor influencing efficacy in these settings.”

When selecting thresholds for recommending where and when chemoprevention strategies should be used, practical factors unrelated to evidence about resistance and efficacy should also be considered, such as whether or not current or future treatment drugs share resistance mechanisms with chemoprevention drugs, or whether a given threshold might result in confusing situations such as different recommendations in adjacent areas with similar malaria epidemiologies that happen to have resistance prevalences just above and below the threshold.

These limitations can be mitigated to some extent by standardizing study designs and coordinating multi-centre trials and pooled analyses, as has been done by consortia that have formed to test and implement some chemoprevention strategies. The WHO recommendations on research priorities can also guide researchers to conduct studies that will yield data useful to policy makers, to the extent that researchers are made aware of and follow such recommendations. For example, a standardized protocol for “Preventive Efficacy Studies (PES)” akin to TES studies is currently being developed by the WHO Global Malaria Programme.

It is somewhat encouraging that malaria chemoprevention does not inevitably lead to meaningful increases in resistance, and even high rates of resistance do not necessarily impair chemoprevention efficacy. At the same time, it can reasonably be anticipated that, over time, as drugs are widely used, resistance will generally increase, and sooner or later efficacy will be lost. Decisions about whether, where and when chemoprevention strategies should be deployed will continue to need to be made on the basis of imperfect evidence. It is hoped that this assessment of what is known about the relationships between resistance and chemoprevention will be useful as the WHO evaluates and updates its chemoprevention recommendations.

## Data Availability

Data sharing not applicable to this article as no datasets were generated or analysed during the current study.

## Abbreviations

ACT: Artemisinin-based combination therapy
AQ: Amodiaquine
DHA: Dihydroartemisinin
DHFR: *Plasmodium falciparum* Dihydrofolate reductase enzyme
*dhfr*: *P. falciparum* Gene that encodes DHFR
DHPS: *P. falciparum* Dihydropteroate synthase enzyme
*dhps*: *P. falciparum* Gene that encodes DHPS
EIR: Entomological inoculation rate
IC50: 50% Inhibitory concentration (measure of in vitro drug resistance)
IPTi: Intermittent preventive treatment in infants
IPTp: Intermittent preventive treatment in pregnancy
ITN: Insecticide-treated net
*k13*: *P. falciparum kelch 13* Gene
MDA: Mass drug administration
*pfcrt*: *P. falciparum* Chloroquine resistance transporter gene
*pfmdr1*: *P. falciparum* Multi-drug resistance gene
*pfpm2/3*: *P. falciparum* Plasmepsin 2/3 gene(s)
RDT: Rapid diagnostic test
RRR: Relative risk reduction
SMC: Seasonal malaria chemoprevention
SNP: Single nucleotide polymorphism (also known as point mutation)
SP: Sulfadoxine-pyrimethamine
WHO: World Health Organization
Chemoprevention: The use of anti-malarial medicines for prophylaxis and for preventive treatment. This review focuses on preventive treatment, and not on prophylaxis for visitors to endemic areas
Prevalence: Prevalence of a given mutation or haplotype is defined here as the proportion of infected individuals in whom that marker or haplotype is detected, irrespective of whether other alleles or haplotypes (e.g., wild-type) are also present in the infection
Quadruple mutant: Dhfr triple mutant plus dhps A437G in the same infection
Quintuple mutant: Dhfr N51I/C59R/S108N and dhps A437G/K540E2 in the same P. falciparum infection. Some publications use “quintuple mutant” (or sextuple or septuple mutant) to refer to any dhfr/dhps haplotype containing any combination of five (or six or seven) mutations, but most use the definition applied here
Sextuple mutant: Dhfr/dhps quintuple mutant plus dhps A581G in the same infection
Triple mutant: Dhfr mutations N51I/C59R/S108N in the same infection
